# Co-morbidity, treatment outcomes and factors affecting the recovery rate of under -five children with severe acute malnutrition admitted in selected hospitals from Ethiopia: retrospective follow up study

**DOI:** 10.1186/s12937-018-0423-1

**Published:** 2018-12-18

**Authors:** Behailu Derseh, Kalayu Mruts, Takele Demie, Tesfay Gebremariam

**Affiliations:** Department of Public Health, College of Health Sciences, Debre Berhan University, P.O. Box 445, Debre Berhan, Ethiopia

**Keywords:** Co-morbidity, Kwashiorkor, Severe acute malnutrition, Treatment outcomes

## Abstract

**Background:**

In spite of the availability of guidelines for the management of severe acute malnutrition (SAM) in Ethiopia, high comorbidity and poor treatment outcomes are still observed in therapeutic feeding centers among under -five children with SAM. The aim of this study was to assess comorbidity, treatment outcomes and factors affecting the recovery rate of children aged 1–59 months with SAM admitted into Therapeutic Feeding Centers (TFCs).

**Methods:**

A total of 413 records of children with SAM admitted in three selected hospitals from July 2013 to July 2015 G.C were retrospectively reviewed. Descriptive analysis was used to compare performance indicator values with SPHERE project reference standards (the minimum standard to be attained during nutritional therapy). Cox-proportional hazard regression analysis was performed to estimate the predictors of recovery rate at *p*-value < 0.05.

**Result:**

The mean age of children was 16 months (95% CI, 15.0, 17.0). Out of 413 children with SAM, 231 (55.9%) recovered, 24 (5.8%) died and 16.3% were defaulted from TFCs. The mean weight gain (in gram per weight of kilogram per day) for recovered children was 15.61 g/kg/day (95% CI, 14.15, 17.07). The overall median recovery time for these children was 12 days (95% CI, 11.22, 12.78). Moreover, most (77.5%) of children admitted with SAM were marasmic followed by Kwash (16%). Pneumonia (54.8%), diarrhea (41.8%) and rickets (21.4%) were co-morbidities which affected SAM children. A child being admitted at Mehal Meda Hospital (Adjusted Hazard Ratio (AHR) = 2.01; 95% CI: 1.34, 2.91), edematous form of malnutrition (AHR = 0.59; 95% CI: 0.39, 0.90) and being a child infected with pneumonia (AHR = 0.71; 95% CI: 0.51, 0.98) were predictors of nutritional recovery rate.

**Conclusion:**

Under five pneumonia, diarrhea and rickets were co-morbidities that should be prevented. Recovery rate was poor when compared to SPHERE project reference value (which is > 75%). Predictors, namely presence of pneumonia and edematous form of malnutrition reduced nutritional recovery rate. Whereas, being admitted at Mehal Meda Hospital improved recovery rate. Therefore, hospitals should work in collaboration with health extension workers to prevent co-morbidities and strengthen screening and referral of malnutrition cases at community level. Moreover, Zonal Health Department and District Health Offices should facilitate experience sharing among health facilities.

## Introduction

Malnutrition is an abnormal physiological condition caused by deficiencies or imbalances in energy, protein and/or other nutrients. It is also explained as “a state in which the physiology of an individual is impaired to the point where he/she can no longer maintain adequate bodily performance, like growth and recovering from disease” [1–3]. According to World Health Organization, Severe Acute Malnutrition (SAM) is defined as a Weight-for-Height value < − 3 Z scores or Middle Upper Arm Circumference (MUAC) < 115 mm or children having bilateral pitting edema. This form of malnutrition is caused by poor-quality diets, weak care for mothers and children, insufficient access to health services, and unsanitary or unhealthy environments [3]. Severe acute malnutrition is the common cause of morbidity and mortality among under-five children all over the world and with the highest concentration in Sub Saharan Africa and South-East Asia [1–3]. According to 2017 global nutrition report, 52 million children are wasted (SAM) and it is the main challenge to achieve the sustainable development goals [4]. Moreover, it is a risk factor for more than 50% of 11 million deaths annually and the number-one driver of the global burden of disease [5, 6].

Severely malnourished children typically are brought to medical attention when a health crisis, such as an infection, precipitates the transition between marasmus (a state of nutritional adaptation) and kwashiorkor, in which adaptation is no longer adequate [5–7]. However, the clinical assessment of the child with malnutrition includes distinguishing between marasmus and kwashiorkor, assessing the severity of malnutrition, and identifying acute life-threatening complications, including sepsis, acute dehydration, and other complications. These children are also at risk for micronutrient deficiencies. An intensive and comprehensive approach is required to reduce the mortality rate associated with this condition and improvement in recovery rate is compulsory [7].

Considering the high burden of the problem, the seriousness and complicated condition, and the management and outcome, children with severe acute malnutrition should be properly and promptly treated in a suitable health facility at therapeutic feeding centers. It was seen that over the last several decades of the twentieth century, the median case fatality rate in developing countries remained unacceptable and relatively higher rates for edematous cases [8]. Despite the efforts made so far, Ethiopia has a high burden of under-five malnutrition (stunting 38%, underweight 24% and wasting 10%) which is an indication of the high magnitude of severe acute malnutrition in the country. Beyond its long-term preventive measures, the country has established different therapeutic feeding centers that can reduce under-five morbidity and mortality attributed to SAM [9].

Several studies have been reported on the magnitude of co-morbidity with SAM which ranges from 1.9% (hypothermia) to 72.9% (diarrhea) and recovery rate which ranges from 60.4 to 87% [10–16]. Co-morbidity, health facility, forms of malnutrition and age were factors associated with recovery rate among under-five children with SAM in Some TFCs of selected hospitals in Ethiopian and elsewhere. These findings in general showed inconsistent evidence across different geographic areas and time of events [10–16]. Besides, studies done so far in Ethiopia are limited to show all components of treatment outcomes and inadequate to compare treatment outcomes from adjacent health facilities.

Furthermore, since the establishment of therapeutic feeding centers in Debre Berhan referral Hospital, Enat general and Mehal Meda primary hospitals, data from therapeutic feeding centers was not analyzed or interpreted. But it is evident that comprehensive data analysis helps to evaluate the effectiveness of TFCs and identify determinant factors that affect treatment outcomes of TFCs. Therefore, the purpose of this study was to assess comorbidity status, treatment outcomes and to identify factors affecting the recovery rate of under-five children with SAM in TFCs. The result of this study also will be used as an input for improving the management of severe acute malnutrition in the selected TFCs and other similar settings in Ethiopia.

## Methodology

### Study design and settings

Retrospective follow up study was conducted at Debre Berhan referral hospital, Enat general and Mehal Meda primary hospitals. Debre Berhan hospital was the only referral hospital in North Shoa Zone and provides services for 2.4 million people in the catchment area. In these hospitals, Severely Malnourished children are admitted in Therapeutic Feeding Centers (TFCs) and further diagnosed and treated by pediatricians, general practitioners, health officers and nurses [17].

### Study participants and data sources

Study participants were severely malnourished children admitted in TFCs based on the Protocol for the Management of SAM in Ethiopia [18, 19]. These participants were under five children with SAM admitted from July 1, 2013 to July 31, 2015. The admission criteria for participants (whose age > 6 months to 59 months) were Weight/Height value < 70% or Middle Upper Arm Circumference (MUAC) < 110 mm or children having bilateral pitting edema. Similarly, participants less than 6 months could be admitted if they are too weak to suckle breast milk or weight/length < 70% or presence of bilateral edema. Data was collected using data extraction form designed for this specific study from inpatient therapeutic feeding registration book, multi-charts and medical records. A total of 413 children documents were considered in this study after diagnosing the completeness of the records.

The outcome variable called recovery rate from SAM was obtained by computing the number of patients discharged for recovery divided by the total number of discharges. Similarly, time-to-cure from SAM was calculated by subtracting the start of treatment date from the date of cure or censored (in days). Since our statistical analysis technique was survival analysis, we recoded treatment outcomes into two categories: Event (Recovery Rate = 1) and all the remaining outcomes considered as censored (Censored = 0). Cured children according to SAM treatment protocol are defined as children who have a weight- for- height > 85% and no bilateral edema. Children were considered to be cured and discharged, which is our event of interest if they fulfilled the discharging criteria for SAM [15]. However, the time-to-cure were censored for those children transferred to other wards, dropped treatment, died, did not cure at the end of the study. The following variables were considered for their influence on the recovery rate from SAM; age, sex, health facility, edema, Naso-gastric tube insertion, and co-morbidity. For age, we used three categories; Less than 12 months, 12–24 months and greater than 24 months. Health facilities had three categories; Debre Berhan Referral, Enat and Mehal Meda Hospitals. Bilateral edema was categorized based on whether the child had an edematous form of malnutrition or not. Co-infection was categorized based on whether the child has coinfection such as anemia, pneumonia, measles and giardiasis or not. The phrase, performance indicator used to mean the character which monitors the performance of TFC and indicates the quality of care provided in terms of attributes (Cure rate, death rate, defaulter rate and etc.) [20].

### Data collection and quality assurance

A structured data extraction form was designed to collect data from the registration book, inpatient multi -chart, and patient medical records. Patient’s medical record number was obtained from therapeutic feeding registration book, so that their records were easily retrieved from medical record units to predict the survival status and the outcome of interest between July 2013–2015.

Data was collected by BSc nurses who had been working in the same health facilities after training on the techniques of data collection. Prior to actual data collection, a pre-test was carried out to check the functionality of data extraction form. The completeness of data was checked by three trained supervisors so as to provide feedback in the data collection process and to provide appropriate measures when necessary. Furthermore, every day after data collection, data collectors, supervisors and principal investigators discussed on quality of data and exchanged information to increase the validity and consistency of the data.

### Data processing and analysis

Data were entered, cleaned and checked for the completeness using EpiData software version 3.1. Errors related to inconsistency were verified using data exploration techniques. Then, data were exported to Statistical Product for Service Solution (SPSS) version 16 software (Release 16.0.0, September 13, 2007, Copyright (c) SPSS, Inc., 1989_2007). Subsequently, data were recoded and checked to facilitate analysis. The Shapiro-Wilk’s test (*p* > 0.05) and a visual inspection of histogram, normal Q-Q plot, normal P-P plot and Box and Whisker plot were used to check the normality of distributions for continuous variables. Descriptive statistical analysis was done using percentages for categorical data and mean/median for continuous variables. Recovery rate, defaulter rate, non-responder rate and case fatality rate of children with SAM were computed. Average weight gain and median length of stay were calculated. Treatment outcomes and other performance indicator values were compared with the SPHERE project reference standards as described in the 2007 SAM management protocol of Ethiopia [19]. The SPHERE standards for performance indicator were the minimum standards for food security, nutrition and food aid that follow the principles and rights of humanitarian charter [20]. Additionally, survival probability was estimated using the Kaplan-Meier method and survival curves were verified by Log-Rank test. Cox-proportional regression analysis was used to measure the association of each independent characteristic with recovery rate. Factors that were associated with time to recovery at 25% [21] significance level in the “Univariate” analysis were included in the final multiple analysis. Statistical significance was declared at *p*-value less than 0.05.

## Results

### Socio -demographic characteristics of SAM children

Out of 413 children with SAM, 229 (56%) were males and the majority (70.8%) of these children was in the age group of 6 to 24 months with the mean age of 16 months (95% CI, 15–17.1). The majority (71.7%) of participants was from Debre Berhan Referral Hospital and 93.7% were Amhara in Ethnicity.

### Medical condition and complications

Of the admitted children with SAM, most (77.5%) had Marasmus and 66(16%) had Kwashiorkor. Concerning co-morbidity, almost all (94%) of admitted children had at least one medical conditions or complications with pneumonia 54.8% followed by diarrhea (41.8%) and Rickets 21.4% “Table 1”*.*

**Table 1:** Co-morbidity and medical conditions among study participants in TFCs of Debre Berhan Referral, Enat General and Mehal Meda Primary Hospitals, 2013–2015

Medical conditions	N (%)
Type of Malnutrition	
*Marasmus*	320 (77.5)
*Kwashiorkor*	66 (16.0)
*Marasmic Kwashiorkor*	27 6.5)
Admission type	
*New*	383 (92.7)
*Readmission*	30 (7.3)
Edema at admission	
*Present*	89 (21.8)
*Absent*	320 (78.2)
Grade of edema at admission	
*Plus one (+)*	28 (32.2)
*Plus two (++)*	29 (33.3)
*Plus three (+++)*	30 (34.5)
Breast fed during stay	
*Yes*	246 (71.5)
*No*	105 (28.5)
Immunization status	
*Fully vaccinated*	184 (63.4)
*On immunization*	63 (21.7)
*Partially immunized*	23 (7.9)
*Not immunized*	20 (6.9)
Co-morbidity	
*Present*	378 (94.0)
*Absent*	24 (6.0)
Major co-morbidities	
Tuberculosis	20 (5.3)
Pneumonia	207 (54.8)
HIV/AIDS	10 (2.6)
Anemia	62 (16.4)
Diarrhea	158 (41.8)
Rickets	81 (21.4)
Hypothermia (Temp < 35 °C)	13 (3.3)
Fever (Temp > 38.5 °C)	24 (6.2)

### Management of medical complications

The study presented that different clinical management was made to improve the health status of children. Among these, a parenteral antibiotic was given to 88.8% of the children, nasogastric tube and intravenous infusions were given for 55.5 and 15.3% of children with SAM, respectively. Nearly 10% of children also received blood transfusion.

### Treatment outcomes

Concerning the treatment outcomes of children with SAM at discharge, 231 (55.9%) recovered, 24 (5.8%) died, 16.3% defaulted from TFCs. Moreover, the mean weight gain for recovered children was estimated to be 15.6 g/kg/day. The overall performance indicators for respective hospitals were displayed below with its SPHERE project reference values “Table 2”.

**Table 2:** Performance indicators of TFCs of Debre Berhan Referral, Enat General and Mehal-Meda Primary Hospitals as compared to SPHERE project reference values, 2013–2015

Performance indicators	Performance by Health facilities			Overall performance	The SPHERE project reference values	
	Debre Berhan RH	Enat General hospital	Mehal Meda General hospital		Acceptable	Alarming
Recovery rate	51.7%	58%	79.2%	55.9%	> 75%	< 50%
Death rate	6.4%	4.3%	4.2%	5.8%	< 10%	> 15%
Defaulter rate	19.6%	10.1%	4.2%	16.3%	< 15%	> 25%
Mean weight gain	14.5	22.2	16.3	15.6	≥8 g/kg/day	< 8 g/kg/day
Mean length of stay	13.8	13.3	10.6	13.2	< 28 days	> 42 days

### Descriptive survival analysis

In the first week of admission, only 11 (3%) of children were recovered while 132 (43%) within 2 weeks. The percentage of recovery within 3 weeks of admission was estimated to be 53%. The median recovery time was 15 days (95% CI: 14.09, 15.908) “Table 3”.

**Table 3:** Actuarial Life table analysis of children with SAM in TFCs of Debre Berhan Referral, Enat General and Mehal Meda Primary Hospitals, from 2013 to 2015

Interval start time (in days)	Subjects entered interval	Subjects censored	Subjects exposed to risk	Subjects recovered	Probability of recovery
0–7	413	57	384.5	11	0.03
7–14	345	74	308	132	0.43
14–21	139	31	123.5	66	0.53
21–28	42	14	35	12	0.34
28–35	16	4	14	7	0.50
35–42	5	1	4.5	1	0.22
42–49	3	1	2.5	1	0.40
> 49 days	1	0	1	1	1.00

### Kaplan Meier analysis of nutritional recovery time

The overall median recovery time was 12 days (95% CI: 11.22, 12.78), whereas the median recovery time for edematous and wasted children were 16 days (95% CI: 14.27, 17.73) and 11 days (95% CI: 10.21, 11.79), respectively.

### Predictors of recovery rate among under- five children with SAM

After controlling for confounding factors, being treated at Mehal Meda hospital, an edematous form of malnutrition and presence of pneumonia remained the predictors of recovery rate. Children treated in Mehal Meda Hospital were 2 times more likely to recover as compared to those children treated in Debre Berhan Referral hospital (AHR = 2.01; 95% CI: 1.34, 2.91), edematous children were 41% less likely to recover as compared to children without edema (AHR = 0.59; 95% CI: 0.39, 0.90). Additionally, children who had pneumonia were 29% less likely to recover as compared to children who had no pneumonia (AHR = 0.71; 95% CI: 0.51, 0.98) “Table 4”.

**Table 4:** Univariate and Multiple analyses for predictors of recovery rate for children with SAM in TFCs of selected hospital, Ethiopia, 2013–2015

Covariates	Recovered	Censored	Crude HR (95% CI)	*p*-value	Adjusted HR (95% CI)
	Frequency	Frequency			
Age group					
Less than 12 months	102	85	1		1
12–24 months	92	68	0.73 (0.55, 0.97)	0.032*	0.78 (0.53, 1.14)
More than 24 months	27	27	0.85 (0.55, 1.29)	0.440	1.08 (0.62, 1.79)
Health Facility					
Debre Berhan RH	153	136	1		1
Enat General H	40	25	1.60 (1.12, 2.28)	0.009*	0.91 (0.47, 1.72)
Mehal Meda PH	38	7	1.80 (1.29, 2.50)	0.000*	2.01 (1.34, 2.91)**
Complementary fed					
Yes	143	136	1.46 (1.05, 2.01)	0.023*	0.89 (0.57, 1.41)
No	50	16	1		1
Pneumonia					
Present	111	86	0.79 (0.6, 1.04)	0.098*	0.71 (0.51, 0.98)**
Absent	95	72	1		1
Anemia					
Present	28	23	1.34 (0.89, 1.99)	0.155*	0.94 (0.61, 1.46)
Absent	177	125	1		1
Rickets					
Present	51	28	0.683 (0.49,0.94)	0.020*	1.41 (0.98, 2.04)
Absent	151	127	1		1
NG tube inserted					
Yes	103	97	1.26 (0.95, 1.66)	0.112*	0.84 (0.60, 1.17)
No	94	60	1		1
Bilateral edema					
Present	42	188	0.50 (0.35, 0.71)	0.000*	0.59 (0.39, 0.90)**
Absent	47	132	1		1

N.B: (*) indicates that significant variables in Univariate analysis at 25% level of significance

(**) indicates that significant variables in Multiple analysis at 5% level of significance

## Discussion

This study was intended to assess the co-morbidity status, treatment outcomes and factors affecting nutritional recovery rate of children with SAM treated in TFCs of three hospitals from Ethiopia. The main findings indicated that in spite of using updated guideline recommended for inpatient management of severely malnourished under -five children [19, 22], co-morbidities were impairing nutritional recovery, treatment outcomes were poor and predictors, like pneumonia, edematous form of malnutrition reduced recovery rate. Whereas, a child being admitted at Mehal Meda hospital improved recovery rate by two- fold.

Thus, in this study, Marasmus was the most common (77.5%) form of severe acute malnutrition followed by Kwashiorkor (16%) and Marasmic Kwashiorkor (6.5%). Similar findings were reported by different scholars from different parts of Ethiopia, Tanzania and Ghana [23–30]. This implies that in some parts of our study setting the land is degraded and the soil has lower fertility, yielding low crop productivity. In contrast, studies conducted in Southern Ethiopia [16, 31], Malawi [32], Zambia [33] and Uganda [34] reported that the edematous form of malnutrition was the most common. Because of the difference in food security status, feeding practice and socio-cultural factors, it is not unique to have different forms of childhood nutritional problem among different geographic areas.

There was a statistical difference (*p*-value < 0.05) between the median recovery time for edematous children (16 days) and their counterparts (11 days). Furthermore, this study depicted that those children who were diagnosed as wasted were more likely to recover earlier than those children diagnosed with edematous. This could be due to edematous children being prone to developing a metabolic complication and fluid overload with chronic diseases. Moreover, children with bilateral edema are more likely to be diseased than Marasmic children; as a result, their condition is more severe and would take longer to recover [35].

Poor recovery rate (55.9%) was projected as one of the treatment outcomes as previously reported from different TFCs in Ethiopia; (such as Felege Hiwot Hospital [25]; Gondar University Tertiary Hospital [26]; Sekota Hospital [27]; Gedeo Zone [31] and other facilities like Tamale Teaching Hospital from Ghana [30], Allahabad Hospital from India [11] and Hospital from North Uganda [34]. On the other hand, retrospective studies from different part of Ethiopia (Yirgalem hospital [28], Woldia hospital [15], Wolisso hospital [36], Jimma specialized hospital [37], hospitals from Southern region of Ethiopian [10]) and a study in Malawi [32] reported that recovery rate was within an acceptable range as compared to the minimum standard of SPHERE project reference values [20]. This could be due to higher defaulter rate in our study compared to other studies, which could decrease the chance of recovery. Additionally, since one of the study areas (DBRH) being a referral hospital, due to patient overload, high burden of co-morbidities and the late arrival of cases (after the development of complication) might also be the explanation for poor recovery rate.

The study showed that severely malnourished children who were treated at Mehal Meda TFC had significantly higher nutritional recovery rate than children treated in Debre Berhan TFC. This was consistent with other findings where nutritional recovery rate might be different among TFCs as explained by Teferi et al. [10], Gebremichael [16], Grum T et al. [31], Chiwaula Maggie [32] and Ngallaba Sospatro et al. [38]. This could be due to malnourished children coming to TFC and being admitted after a complication had occurred. Since DBRH_TFC is used as referral health institution, cases may have come in increased severity, which was not the case for Mehal Meda TFC. In addition, this result could be due to the increased caseload of SAM since more children were admitted at Debre Berhan referral hospital than Mehal Meda TFC. In general, we can explain the difference in terms of difference in study settings, case load and severity of cases.

This study also showed that children with SAM who developed pneumonia had a significantly lower nutritional recovery rate than children without pneumonia. This could be explained in terms of the synergistic relationship between pneumonia and malnutrition, which has been well recognized by Cervantes and San [39]. Moreover, children with pneumonia may have an increased respiratory rate and use of accessory muscles of respiration thereby needing to stay longer in therapeutic feeding centers to recover. In addition, the presence of co-morbid conditions characterized by inadequate food intake may lead to fast depletion of nutrients and delayed nutritional recovery.

Increasing evidence suggests that malnutrition is the underlying reason for the increased susceptibility to pneumonia which results in a vicious cycle [35]. This indicates that hospitalized children experiencing malnutrition need an early diagnosis of co-morbidity and prompt treatment before developing a complication, many of which make them more vulnerable and reduce the chance of nutritional recovery. Similar findings were observed in Tanzania [29]. In our study, 94% of the cases developed at least one form of co-morbidity, which may have been due to their poor immunity to prevent nosocomial infection. Additionally, it may also be due to small bowel bacterial overgrowth and parasitic infections that commonly occur in SAM cases. These children are also the leading harbors of parasites that directly consume nutrients and prevent nutrient absorption [36]. This also explains the decrease in nutritional recovery rate in the present study.

Overall, this study is able to show co-morbidities, treatment outcomes and predictors of recovery rate among under-five children with SAM in three selected TFCs of North Shewa Zone, Amhara regional State of Ethiopia. Since these three TFCs are catchment areas for millions of people in the zone, it has paramount importance in recognizing the performance of TFCs and take remedial actions for better survival.

In spite of its strength, this study has limitations. It used staffs working in the same therapeutic feeding centers as data extractors from records, inpatient multi- charts, and medical records. However, to avoid subjectivity most of the variables reviewed as associated factors for treatment outcomes of SAM were objective in type. Moreover, since the study fully relied on secondary data, it is impossible to include essential risk factors that could have further explained the association among independent factors and recovery rate.

## Conclusion and recommendations

In conclusion, nutritional recovery rate in our study is poor when compared to the SPHERE project reference value. Additionally, the defaulter rate in the Debre Berhan referral hospital TFC needs to be improved. Regarding other performance indicators, mortality rate, mean weight gain and mean length of hospitalization were in the acceptable range of international standards in all three hospitals. This study summarizes the presence of pneumonia, the presence of edema reduced nutritional recovery rate whereas a child being admitted at Mehal Meda Hospital TFC improved recovery rate. Moreover, this study presents the high rate of co-morbidity that hinders nutritional recovery rate. Therefore, to prevent complications, improve recovery rate and decrease defaulter rate, emphasis should be given to improving early detection and treatment of severely malnourished children in the catchment areas. This can be done by creating a strong community- based screening and referral of malnutrition cases with the support of health extension workers. In addition, Zonal Health Department and District Health Offices should support and coordinate experience sharing among health facilities. Finally, combating childhood illness particularly pneumonia, diarrhea, rickets and to detect malnutrition early before developing complications should be done.

## Abbreviations

AHR: Adjusted hazard ratio
CI: Confidence interval
DBRH: Debre Berhan Referral Hospital
ICU: Intensive care unit
IMNCI: Integrated maternal, neonatal and childhood illness
SAM: Severe acute malnutrition
SPSS: Statistical package for social sciences
TFC: Therapeutic feeding center
